# Passive Vaping from Sub-Ohm Electronic Cigarette Devices

**DOI:** 10.3390/ijerph182111606

**Published:** 2021-11-04

**Authors:** Maurizio Manigrasso, Carmela Protano, Matteo Vitali, Pasquale Avino

**Affiliations:** 1 Department of Technological Innovations, National Institute for Insurance against Accidents at Work (INAIL), 00187 Rome, Italy; 2 Department of Public Health and Infectious Diseases, Sapienza University of Rome, 00185 Rome, Italy; matteo.vitali@uniroma1.it; 3 Department of Agricultural, Environmental and Food Sciences (DiAAA), University of Molise, 86100 Campobasso, Italy; avino@unimol.it

**Keywords:** electronic cigarette, sub-ohm, aerosol, passive vaping exposure, atomizer geometry, size number distribution

## Abstract

To investigate passive vaping due to sub-ohm electronic cigarettes (e-cigs), aerosol number size distribution measurements (6 nm–10 µm) were performed during volunteer-vaping sessions. E-liquids, with vegetable glycerin (VG) and propylene glycol (PG), with a VG/PG ratio of 50/50 (with nicotine) and 80/20 (without nicotine), were vaped with a double-coil, single aerosol exit hole at 25–80 W electric power, corresponding to 130–365 kW m^−2^ heat fluxes and with an octa-coil, four aerosol exit holes atomizers, at 50–150 W electric power, corresponding to 133–398 kW m^−2^ heat fluxes. At the lowest heat flux, lower particle number concentrations (N_Tot_) were observed for the nicotine-liquid than for the nicotine-free liquid, also due to its higher content of PG, more volatile than VG. For the octa-coil atomizer, at 265 and 398 kW m^−2^, N_Tot_ decreased below the first-generation e-cig, whereas volume concentrations greatly increased, due to the formation of super micron droplets. Higher volume concentrations were observed for the 80/20 VG/PG liquid, because of VG vaporization and of its decomposition products, greater than for PG. For the double coil atomizer, increasing the electric power from 40 W (208 kW m^−2^) to 80 W (365 kW m^−2^) possibly led to a critical heat flow condition, causing a reduction of the number concentrations for the VG/PG 50/50 liquid, an increase for the 80/20 VG/PG liquid and a decrease of the volume concentrations for both of them. Coherently, the main mode was at about 0.1 µm on both metrics for both liquids. For the other tests, two main modes (1 and 2 µm) were observed in the volume size distributions, the latter becoming wider at 100 and 150 W (265 and 398 kW m^−2^), suggesting the increased emission of light condensable decomposition products. The lower aerosol emissions observed at 150 W than at 100 W suggest the formation of gas-phase decomposition products. The observation of low-count high-volume aerosols addresses the relevance of the volume metric upon measuring the second-hand concentration of the aerosols released by sub-ohm e-cigarettes.

## 1. Introduction

The term electronic cigarette, also called e-cig, is used to indicate different devices allowing the users to inhale an aerosol, which can contain nicotine, flavorings, and other additives [1,2].

From its introduction to the market, e-cigs varied widely in design and appearance. In general, they operate in a similar manner and consist of three components: a battery, an electrical heater, and a liquid that is aerosolized for users to inhale [3]. However, in the course of the years, their characteristics have evolved. Even if a systematic classification and a standardized terminology are lacking [4], researchers commonly recognize four different e-cig generations [5,6,7,8,9]. First-generation e-cig or cig-a-like/cartomizer products are similar in size and design to a traditional cigarette, second-generation e-cig or clearomizers presents larger atomizers and/or tanks, third-generation devices have larger fluid reservoirs and batteries and can be used with various power settings, and fourth-generation e-cigs present relatively low-powered batteries and can be used with sub-ohm resistances, allowing to vape at higher power settings [9]. Moreover, the term “fourth-generation” is also used to indicate the so-called pods (i.e., JUUL) [6,7].

From the first commercialized e-cig to the currently widely used fourth-generation devices, scientific evidence on related acute and chronic adverse effects on human health has accumulated [10]. A recent review highlighted that the use of e-cigs determines negative changes in cardiovascular, respiratory, and immune systems, increasing blood pressure, heart rate, arterial stiffness, and resistance to air flow in lungs and causing the production of immunomodulatory cytokines. Results from animal and in vitro studies confirmed that long-term exposure to e-cigs is associated to significant health risks for these systems [11]. Moreover, since 2019, a new epidemic of a syndrome called e-cigarette or vaping product use-associated lung injury (EVALI) has affected thousands of U.S. individuals [12]. It is well known that a fraction of e-cig aerosol emitted reaches pulmonary alveoli; at that site, the volume of liquid that get dissolved into the thin (reported thickness vary from 20 nm [13] to 0.1 µm [14]) surfactant film that covers the alveolar surface represents a relevant parameter. In relation to that, it has been recently suggested [15] that chronic inhalation of e-liquid solvents may play a role in EVALI by altering the function of the pulmonary surfactant. Other researchers reported also a link between e-cig use and cancer development and growth through several mechanisms [16,17]. E-cig-related health problems can involve not only vapers, but also passive exposed to the aerosol emitted during the e-cigs use. Indeed, recent studies demonstrated the presence of various toxics [9], Particulate Matter (PM) [18], and Ultrafine Particles (UFPs) [19] in the aerosol emitted by e-cigs, and their levels differ depending on several factors, including the technical features of the devices [9,18,19].

These findings are of great concern for public health, because the use of e-cigs has been dramatically increasing worldwide, with high prevalence in North America (5.5% of adults and 5.2% of youth, respectively), England (5.5% of adults and 2.2% of youth, respectively), and Poland (35% among adolescents) [10]. The Centre for Disease Control and Prevention (CDC) indicates that, currently, e-cigs are the most commonly tobacco product used by adolescents and, in 2020, 3.6 million U.S. middle and high school students have used e-cigarettes over a 30-day period. Moreover, in 2019, 4.5% of U.S. adults were current e-cig users (also called vapers) and 36.9% of these individuals were dual users (currently vapers and traditional smokers) [20].

Within this context, the purpose of this study is to compare the first commercialized cig-a-like/cartomizers to sub-ohm fourth-generation e-cigs and to investigate to what extent passive vaping exposure has been affected.

## 2. Materials and Methods

### 2.1. E-Cigs and the Experimental Plan

Aerosol measurements have been carried out using commercial e-cig liquids and resistance coils selected form those most commonly used at the time of the experiments. Experimental conditions were selected to reproduce real-scenario vaping conditions. In particular, a first-generation and a fourth-generation e-cig together with the liquids were identified through a simple market analysis as those most used among vapers. Identified devices and liquids were used for the experiments.

The first-generation e-cig used during the tests was the young category (Categoria, Oleggio (NO), Italy), an e-cig with a disposable filter, integrated atomizer, and cotton already soaked in liquid. Liquids used with the first-generation e-cig were, respectively, a liquid without nicotine (1st e-cig No Nic) and a liquid with a nicotine at 24 mg mL^−1^ (1st e-cig Nic).

The fourth-generation e-cig used during the tests was the G150 Kit (Smok^®^, Shenzhen, China) equipped with three different coils in order to test different performance conditions: Smok^®^ V8 Baby-Q2 (0.4 Ω; usable at 40–80 W—best use between 55–65 W), Smok^®^ V8 Baby-Q2 (0.6 Ω; usable at 20–50 W—best use between 30–40 W), and Smok^®^ V8 Baby-T8 (0.15 Ω; usable at 50–110 W—best use between 60–80 W). The liquids used were: Pacha Mama—Mango Pitaya Ananas^®^ without nicotine and with vegetable glycerin (VG) and propylene glycol (PG), with a VG/PG ratio of 80/20, and SmookeKannel^®^ with nicotine at 9 mg mL^−1^ and with a ratio VG/PG equal to 50/50.

Table 1 shows the main characteristics of the coils and the relevant wattage tested, together with the relevant heat fluxes, used with the fourth-generation e-cig. Heat fluxes (kW m^−2^) have been calculated as the ratio of electrical power and the heating coil surface area [21].

In total, a set of 14 experimental tests were performed and repeated in triplicate. The sequence was defined generating a randomization list with blocking based on the device and e-liquid type. Each test consisted of a vaping session performed by the same vaping volunteer using different devices, liquids, and electrical parameters. The devices, the e-liquids characteristics, the operative conditions, and the relevant codes of the experiments carried out are summarized in Table 2.

The ranges of nicotine levels and the electrical parameters were those typically used by vapers.

Each vaping session consisted of 12 puffs (1 puff every thirty seconds for a total of 5.5 min calculated by using a chronometer) in the same way as traditional cigarettes (10–12 puffs of a cigarette for a period of about 5–6 min) [22]. All the tests were carried out by the same vaping volunteer, who was already a smoker and familiar with both devices used in the study, and employed at the University of Rome La Sapienza. The study was conducted in compliance with the guidelines of the Declaration of Helsinki, and approved by the Local Ethics Committee (Azienda Ospedaliero Universitaria Policlinico Umberto I of Rome, protocol code n. 3520).

### 2.2. Aerosol Measurements and Quality Assurance

Aerosol measurements were carried out in a 53 m^3^ test room. The room air exchange rate, 0.63 h^−1^, was measured by the tracer gas technique [23], as reported in Manigrasso et al. [19]. Throughout the aerosol measurements, temperature and relative humidity were 21–23 °C and 54–58%, respectively. The room was ventilated after each experiment, opening the door and window to rebalance the room atmosphere for at least three hours and, in any case, long enough to reach the aerosol background level.

Aerosol size number distributions were measured with 1-s time resolution [24] as functions of electrical mobility diameter, in the range 5.6–560 nm, with 32 size channels, by means of a fast mobility particle sizer (FMPS, model 3091, TSI, Shoreview, MN, USA). The performances of FMPS were checked by comparison with a Scanning Mobility Particle Sizer (SMPS, model 3936, TSI) equipped with an Electrostatic Classifier (model 3080, TSI), a Differential Mobility Analyzer (DMA, model 3081, TSI), and a Condensation Particle Counter (model 3775, TSI). The FMPS number concentrations were approximately 15% lower than the diffusion loss corrected SMPS number concentrations, in agreement with the findings of Jeong and Evans [25].

In the range from 0.3 to 10 µm, measurements were carried out with 1-s time resolution as functions of optical diameter, by means of an Optical Particle Sizer (OPS, model 3330, TSI). OPS calibration was carried out with polystyrene latex (PSL) [26]. Size errors, due to aerosol refractive index different from PSL refractive index, were minimized by means of the OPS Mie scattering calculation capability. To this end, the refractive index was set to 1.43, as measured for a range of e-cig liquids by Pratte et al. [27].

FMPS and OPS size number distribution were merged fitting the data point in the overlapping region by a power-law Junge distribution [28] (Equation (1)):

(1) $$\frac{dN}{dlogD}=CD^{-k}$$

where *N* is the number concentration, *D* is the particle diameter, and *C* and *k* are constant retrieved by a least square fitting procedure.

Volume size distributions were calculated from the relevant number size distribution, considering aerosols are made of spherical droplets.

The instruments were placed with their inlets at approximately 1.5 m above the floor and at approximately 1.5 m away from the active vaper to simulate the breathing zone of a passive exposed subject.

## 3. Results and Discussion

**Figure 1:**
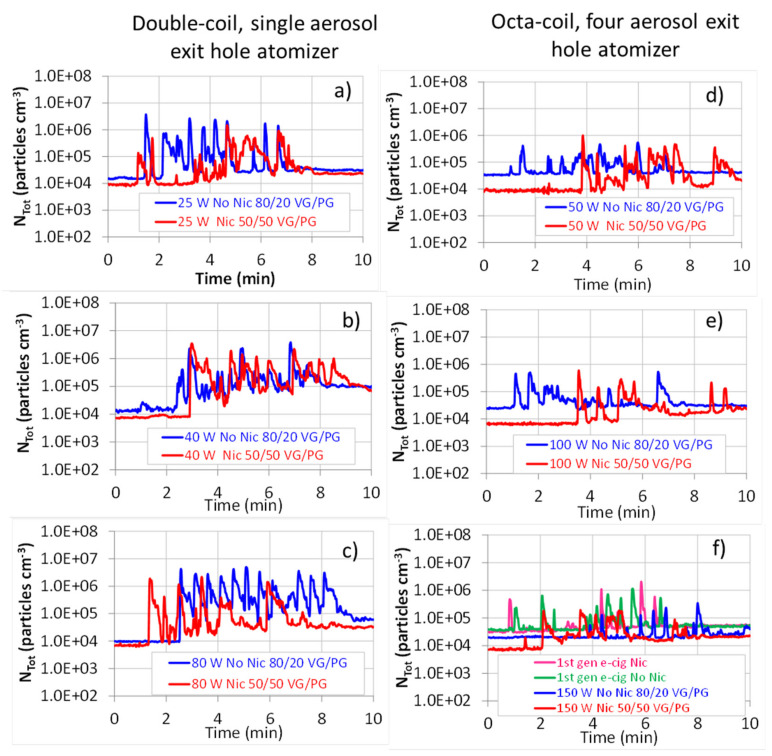
Temporal trend of the total particle number concentration (N_Tot_) during the vaping sessions of fourth-generation e-cigarette. N_Tot_ peaks correspond to the volunteers’ puff exhalations. Double-coil, single aerosol exit hole atomizer at (**a**) 25 W (130 kW m^−2^), (**b**) 40 W (208 kW m^−2^), and (**c**) 80 W (365 kW m^−2^). Octa-coil, four aerosol exit holes atomizer at (**d**) 50 W (133 kW m^−2^), (**e**) 100 W (265 kW m^−2^), and (**f**) 150 W (398 kW m^−2^) and first-generation e-cigarettes.

The vaping sessions at power settings of 25 W, 40 W, and 80 W were carried out with a double-coil, single aerosol exit hole atomizer. The relevant temporal trends of the total particle number concentration (N_Tot_) for nicotine 50/50 VG/PG and nicotine-free 80/20 VG/PG liquids are reported in Figure 1a–c.

At 25 W (130 kW m^−2^) power setting, lower aerosol concentrations were measured for the PG-rich liquid. This is congruent with the vapor pressure of PG (0.13 mmHg at 25°C), lower than the VG one (1.68 × 10^−4^ mm Hg at 25 °C), so that for the lower VG/PG ratio liquid, more solvent partitioned into the vapor phase. Such behavior is in agreement with the study of Li et al. [29], who found that the VG/PG ratio was positively associated with the PM_2.5_ emission factor. Moreover, according to the authors, nicotine also plays a role in reducing aerosol emissions. On that issue, they pointed out the contradictory findings of literature data and studied homemade liquids with different VG/PG ratios. They showed that the addition of nicotine (2.4%) caused a 33% reduction of the particle number emission factor.

Increasing the e-cig power to 40 W (208 kW m^−2^) caused no appreciable variation in aerosol emissions from 80/20 VG/PG liquid, whereas an increase was observed for the more volatile nicotine 50/50 VG/PG. This observation is confirmed by the relevant time number concentrations averaged over the vaping sessions (N^M^_Tot_) reported in Figure S1a. This is congruent with the power-depending liquid consumption described by Jensen et al. [30].

At 80 W (365 kW m^−2^), N_Tot_ decreased for the 50/50 VG/PG liquid, mainly due to the increased PG partitioning into the vapor phase. The formation of high vapor pressure aldehyde, such as formaldehyde and acetaldehyde, should also be considered as a possible cause. On this point, Saliba et al. [31] showed that PG decomposition in air, over different metal wires, occurs with the formation of carbonyl compounds with maximum decomposition temperatures that range from 256 to 460 °C, depending on the carbonyl compound (methylglyoxal, formaldehyde, acetaldehyde, glyoxal, and butyraldehyde) and varying with the metal alloy and the wire aging condition.

On the contrary, N_Tot_ increased for the high VG bearing liquid, suggesting an important VG volatilization, condensing into droplet phase, once cooled at room temperature. Such behavior can be indicative of the attainment of the critical heat flow (CHF) regimen. Under the CHF condition, the resistance temperature increases abruptly, because of the formation of a film of vapor on its surface, which reduces the heat transfer coefficient [32]. The 365 kW m^−2^ heat flux of this study is slightly below the range of 389–662 kW m^−2^, measured by Talih et al. [21] by submerging kanthal wires in a beaker containing PG; however, deviation from such values can be expected, given the differences of the authors’ experimental setting from a real e-cig atomizer.

The 50 W (133 kW m^−2^), 100 W (265 kW m^−2^), and 150 W (398 kW m^−2^) vaping sessions were carried out with an octa-coil, four aerosol exit holes atomizer; the relevant temporal trends of N_Tot_ for 50/50 VG/PG and nicotine-free 80/50 VG/PG liquids are reported in Figure 1d–f.

The relevance of heat flux has been pointed out by Talih et al. [33], who argued that the temperature difference between the heating coil temperature and its surrounding is proportional to the electric power applied per unit coil surface, i.e., to the heat flux. Above all, they showed that the number of aldehydes derived from the thermal decomposition of e-cigarette liquids was well-correlated with the heat flux and poorly correlated with the electrical power. Therefore, increasing the heat flux, by increasing the coil temperature, will also increase the amount of liquid volatilized that, once cooled at room temperature, depending on the vapors pressure, can condense into droplets. The important role played by the coil surface area [21] is clearly described by the N_Tot_ values at 50 W (133 kW m^−2^) power setting, which, due to the higher coil surface area, are lower than those observed for the 40 W (208 kW m^−2^) vaping session, carried out with a double coil single aerosol exit hole atomizer, for both liquids.

**Figure 2:**
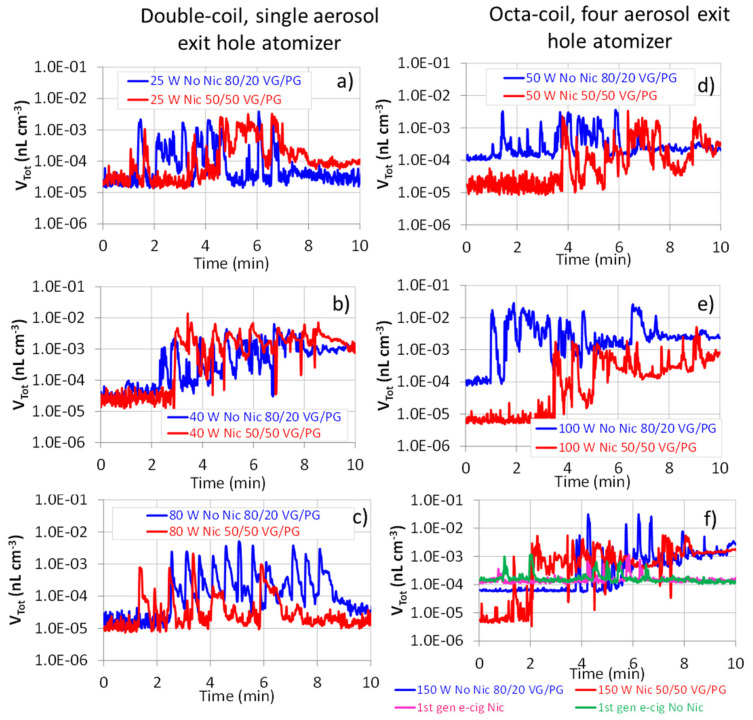
Temporal trend of the total volume aerosol concentration (V_Tot_) during the vaping sessions of fourth-generation e-cigarettes. Double-coil, single aerosol exit hole atomizer at (**a**) 25 W (130 kW m^−2^), (**b**) 40 W (208 kW m^−2^), and (**c**) 80 W (365 kW m^−2^). Octa-coil, four aerosol exit holes atomizer at (**d**) 50 W (133 kW m^−2^), (**e**) 100 W (265 kW m^−2^), and (**f**) 150 W (398 kW m^−2^) and first-generation e-cigarettes.

Increasing the power setting to 100 and 150 W (265 and 398 kW m^−2^, respectively) caused a reduction of N_Tot_, which fell below the values observed for the first-generation e-cig. This occurrence can be understood when expressing the aerosol concentration in a volume metric (Figure 2).

Indeed, the decrease of N_Tot_ at 100 and 150 W (265 and 398 kW m^−2^, respectively) was accompanied by an increase of the total aerosol volume concentration (V_Tot_), which was remarkable for 80/20 VG/PG liquid. Such a circumstance is confirmed by Figure S2, where particle volume concentrations averaged over the vaping sessions (V^M^_Tot_) are reported as functions of the heat fluxes. Increasing the power setting increases the amount of liquid transferred from the e-cigarette wick to the heater coil, which is then vaporized. As the fraction of vaporized PG increases, the liquid becomes enriched in VG and its temperature increases until VG vaporization and thermal decomposition occur. Such vapor condenses, once cooled by the action of the air stream drawn through the e-cig mouthpiece, and the more droplets are generated, the more they undergo coagulation, with consequent reduction in number and increase in volume concentrations. Coherently with that, such an occurrence is remarkable for the 80/20 VG/PG liquid. Another point to explain is why increasing the power setting from 100 (265 kW m^−2^) to 150 W (398 kW m^−2^) causes a clear decrease of the volume concentration for that liquid. We can hypothesize that, at such a high power setting, an important amount of pyrolysis products is light, non-condensable gas-phase products. This interpretation is coherent with the study of El-Hellani et al. [34], who found that, using a sub-ohm e-cig, gases such as carbon monoxide, carbon dioxide, methane, and acetylene were released over 10 wrap dual coil kanthal wires (417 mm^2^ surface area) from a 70/30 VG/PG liquid, at a power setting of 125 W. The authors measured CO concentrations ranging from 77 to 2387 mg m^−3^, depending on the coil alloy and surface area.

**Figure 3:**
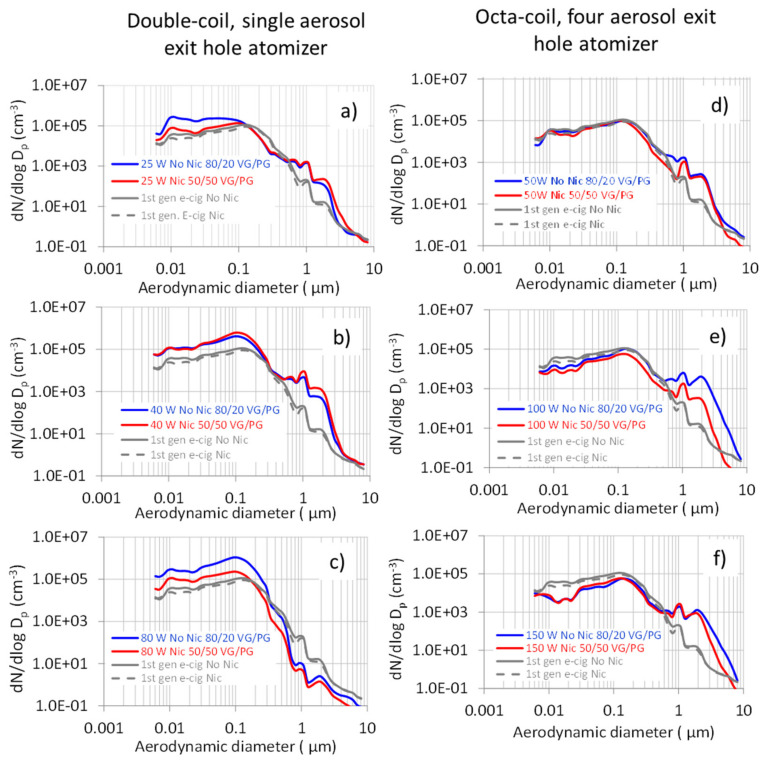
Aerosol number size distributions averaged over the vaping sessions of fourth-generation e-cigarettes, compared with first-generation e-cigarettes. Double-coil, single aerosol exit hole atomizer at (**a**) 25 W (130 kW m^−2^), (**b**) 40 W (208 kW m^−2^), and (**c**) 80 W (365 kW m^−2^). Octa-coil, four aerosol exit holes atomizer at (**d**) 50 W (133 kW m^−2^), (**e**) 100 W (265 kW m^−2^), and (**f**) 150 W (398 kW m^−2^).

**Figure 4:**
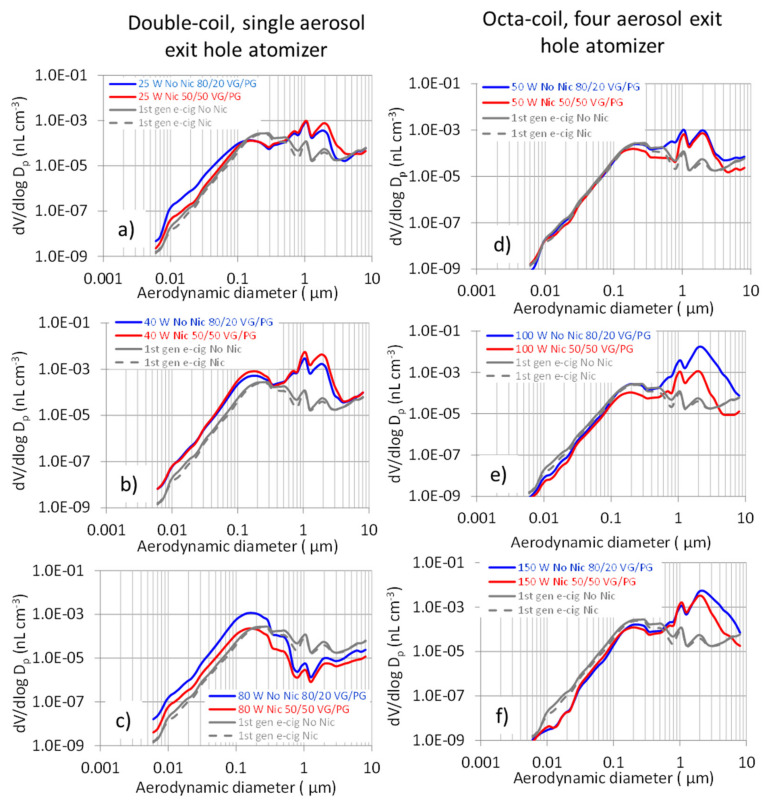
Aerosol volume size distributions averaged over the vaping sessions of fourth-generation e-cigarettes, compared with first-generation e-cigarettes. Double-coil, single aerosol exit hole atomizer at (**a**) 25 W (130 kW m^−2^), (**b**) 40 W (208 kW m^−2^), and (**c**) 80 W (365 kW m^−2^). Octa-coil, four aerosol exit holes atomizer at (**d**) 50 W (133 kW m^−2^), (**e**) 100 W (265 kW m^−2^), and (**f**) 150 W (398 kW m^−2^).

Moreover, comparing Figure 3c–f and Figure 4c–f, it is worth observing the different behavior of the octa-coil atomizer from the double-coil one, although it was operated at a higher heat flow, i.e., 150 W, 398 kWm^−2^, as compared to 80 W, 365 kWm^−2^. We hypothesize that such a difference may possibly be due to the different geometry of the two atomizers, influencing the heat exchange rate and the air flow regimen over the hot metal coil. In particular, we note that the heat transfer coefficient is an increasing function of the air velocity, through the Reynolds number [35], and increasing the air velocity, which then increases the degree of remixing, could also hinder the build-up of the vapor film responsible of the CHF onset over the coil surface.

Moreover, Ooi et al. [36], studying propylene glycol and glycerol mixtures with no other additive, showed that the amount of decomposition products emitted was mainly determined by VG and increased as its concentration increased.

The particle number size distributions averaged over the vaping sessions (Figure 3) showed two main modes at about 0.01 and 0.1 µm, and, at a much lower concentration, a mode at about 1 µm, in agreement with the study of Floyd et al. [37]; moreover, a further minor mode was observed at about 2 µm.

For the double-coil, single aerosol exit hole atomizer, the 0.1 µm mode was more intense at a power setting of 40 W (208 kW m^−2^) and 80 W (365 kW m^−2^) than at 25 W (130 kW m^−2^), due the increased droplet generation and coagulation. Comparatively, the relevant size distributions for the octa-coil four aerosol exit holes atomizer were far less intense, due the lower heat flux (133 kW m^−2^) at a power setting of 50 W and to the lower aerosol exit hole volume, favoring droplet coagulation. Aerosol coagulation explained the lower size distribution observed at 100 W (265 kW m^−2^) and 150 W (398 kW m^−2^), in comparison with the first-generation e-cigarette.

Figure 4 reports the aerosol volume size distributions averaged over the vaping sessions of fourth-generation e-cigarettes compared with first-generation e-cigarettes.

With the exception of the 80 W (365 kW m^−2^) power setting, the relevant volume size distributions (Figure 4) exhibited two intense partially overlapping modes at about 1 µm and 2 µm and a less intense one at about 0.2 µm.

The two main modes are congruent with the lower size number distributions, below the first-generation e-cig, observed for the 100 W (265 kW m^−2^) and 150 W (398 kW m^−2^) power settings, because of the greater aerosol emissions and narrower aerosol exit holes; hence, due to efficient droplet coagulation that gives rise to low number–high volume aerosol. As to the two super-micron modes, we speculate that they were determined by the different densities of the condensed compounds. The heavier compounds, particularly VG (1.26 g cm^−3^ density), would mainly account for the 1 µm mode. The lighter decomposition products, such as butyraldehyde, acrolein methanol, ethanol, and isopropanol (densities in the range 0.78–0.84 g cm^−3^), would allow droplets to grow into larger sizes, before efficient gravitational settling occurs, and therefore, they would possibly be more abundant in the larger 2 µm mode. With this respect, it is worth observing the relevant contribution of VG decomposition to ethanol emissions. Ooi et al. [36] reported that the concentration of this compound in e-cig emissions increases by 4.5-fold PG alone, without added ingredients, to 80/20 VG/PG mixtures, being in a vapor phase as high as 28.6 mg m^−3^. Moreover, the increased release of condensable thermal decomposition products at higher power settings (100 W and 150 W) is evidenced by the 2 µm mode becoming wider and spanning up to about 6 µm.

For the double-coil single air hole atomizers, the 0.15 µm monomodal volume size distribution observed at a power setting of 80 W (Figure 4c), in comparison with 25 W and 40 W vaping sessions, may be explained by the attainment of the CHF regimen.

On the other hand, a similar size distribution was not observed for the 50 W (133 kW m^−2^), 100 W (265 kW m^−2^), and 150 W (398 kW m^−2^) vaping sessions, possibly due to the different geometry of the atomizer, equipped with four air-vapor exit holes, each one accommodating a double coil resistance. Therefore, the flow rate over each double coil resistance was lower in comparison with the double-coil single air-vapor hole configuration. Lower flow rates have been shown to lead to the emission of greater mass median [38] and count median [39] diameter aerosols. Nonetheless, this issue deserves a more specific investigation, since it is expected to markedly affect the aerosol distribution into the human respiratory system.

**Figure 5:**
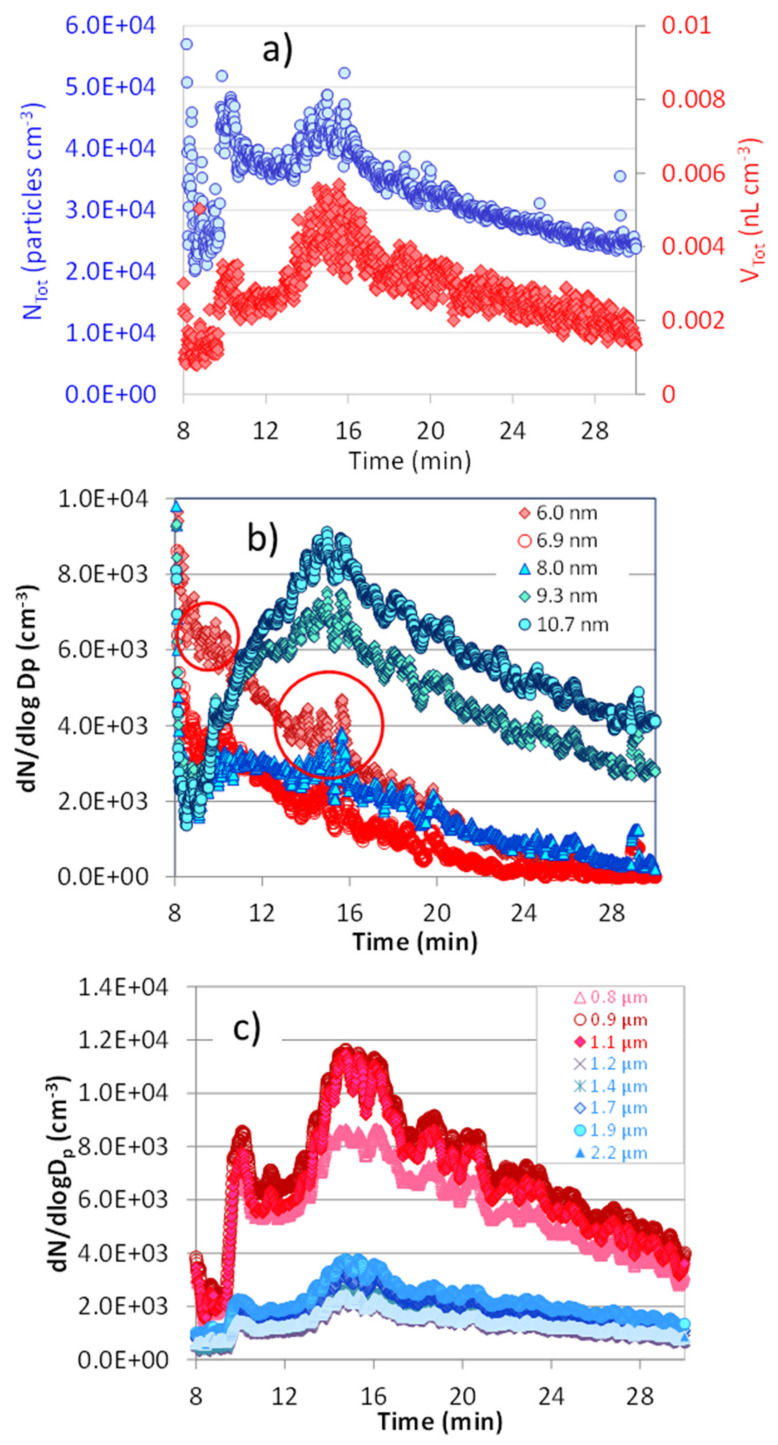
Temporal trend after the vaping session of the nicotine-free 80/20 VG/PG liquid at 150 W (398 kW m^−2^) of (**a**) total number (N_Tot_) and total volume (V_Tot_) of the aerosol concentrations, (**b**) 6–10 nm size fractions, and (**c**) 0.8–2.2 µm size fractions.

Figure 5 shows the temporal trend after the vaping session of the nicotine-free 80/20 VG/PG liquid at 150 W (398 kW m^−2^) with respect to the total number (*N_Tot_*) and the total volume (V_Tot_) aerosol concentrations (Figure 5a), with 6–10 nm size fractions (Figure 5b) and 0.8–2.2 µm size fractions (Figure 5c).

The important role played by vapor phase emissions is evidenced, for 80/20 VG/PG liquid vaped at 150 W (398 kW m^−2^), by the temporal trend of N_Tot_ and V_Tot_, which after the vaping session, increase following two clear maxima (Figure 5a). Figure 5b shows the temporal variation of the number concentration of size fractions in the range 6–10 nm. Data have been smoothed as 20 s moving averages, to clearly show the curve shapes. The 6.0 and 6.9 nm size fractions follow a decreasing trend, due to their efficient diffusion and Brownian coagulation into larger 8–10 nm size droplets that conversely increase their concentrations over time. Coagulation, as such, does not explain the increasing trend of N_Tot_, since it involves a decrease of the total particle number. Indeed, new particles are formed by vapor phase condensation, as shown by the 6.0 and 6.9 nm curves, circled in red in Figure 5b. For those sizes, particle concentrations swiftly increase and decrease due to both coagulation and re-evaporation favored by the small droplet radius (Kelvin effect). Those nucleation episodes are retrieved as two clear maxima in the N_Tot_ and V_Tot_ curves (Figure 5a), in the 8–10 nm (Figure 5b) and 0.8–2.2 µm number concentration curves (Figure 5c). In particular, in Figure 5c two clusters of curves are clearly visible at 0.8–1 µm and at lower number concentrations, but higher volume concentrations (figure not shown), and 1.2–2.2 µm, which corresponds to the bimodal distributions of Figure 4. Figure S3 shows the details of the particle size number and volume distribution measured at the 8, 10-, 16-, 24-, and 30-min time points of Figure 5.

The results presented here confirm that vaping an e-cigs always generate aerosols whose sizes and concentrations depend on the e-liquid composition, as well as on the electrical parameters and the atomizer geometry. Both active vapers and bystanders are exposed to the e-cig’s emissions to an extent that is strongly affected by the wide range of electrical power allowed by the sub-ohm resistance coils. In particular, the abundant presence of 1–2 µm droplets is expected to influence the aerosol deposition into the respiratory system, increasing the amount deposited in the nose, larynx, pharynx, and mouth, in comparison with sub-micron aerosols.

The present study has some limitations. Firstly, the vaping sessions were performed by a real smoker volunteer and, consequently, the results were partially influenced by the individual way of vaping. On the other hand, this approach allows to measure the amount of aerosol truly emitted by a vaper (thus excluding the aliquot retained in the respiratory tract), and not the total aerosol produced by a smoking machine. However, the complete evaluation of the environmental and human health risks related to the e-cigs emissions needs the assessment of their chemical composition and the study of relevant aerosol respiratory dosimetry of those passively exposed. Secondly, we did not carry out a systematic assessment of all the e-cigs models, available e-liquids, and vaping conditions; thus, additional investigations are required to elucidate a more detailed picture of the exposure profile to passive vaping.

## 4. Conclusions

E-cig devices have increased their complexity since the first launch of the first-generation cig-a-like version, where no parameter was adjustable by the users. The last-released and currently used fourth-generation e-cigs allow users to personalize the way they vape, not only with a wide choice of liquids but also by the selection of electrical parameters. Specifically, the possibility to mount sub-ohm resistances has opened the way to considerably increase the amount of liquid vaped per unit puff, with detriment not only of the health of the users but also of the air quality of indoor environments. This study has shown that the atomizer geometry (octa-coil four aerosol exit holes vs. double coil single hole atomizers) and high power settings of 100–150 W (heat fluxes, 265–398 kW m^−2^) cause the emission of droplets lower in number but larger in volume, so that relying only on number-metric aerosol measurements may induce an underestimation of indoor air contamination. Volume-metric aerosol measurements are, therefore, relevant to evaluate the second-hand aerosol concentrations deriving from sub-ohm electronic cigarette devices. The increasing temporal trends of the aerosol number and volume concentrations after the vaping sessions, particularly with the higher VG concentration liquid at the highest power settings, emphasize the role of vapor phase emissions, evidencing the formation of new droplets deriving from vapor condensation. Moreover, we hypothesize that the presence of two main partially overlapping modes at about 1 and 2 µm in the volume size distributions is a sign of aerosol droplets growing up to different limit sizes at which efficient gravitational settling occurs, depending on their densities. The lower density decomposition products are expected to contribute mainly to the lager mode, as indicated by its width becoming broader at the highest power settings. The lower aerosol emissions at 80 W (365 kW m^−2^) and 150 W (398 kW m^−2^), lower than at 100 W (265 kW m^−2^) are the consequence of the release of non-condensable gas-phase products at the highest heat fluxes.

The results obtained demonstrate that the sub-ohm e-cigs determine a worsening of the indoor air quality, during and after their use, because of the wide range of electrical power allowed. Consequently, specific normative should be enforced to regulate the use of e-cigs in public indoor environments, while educational interventions are needed for increasing the vapers’ perception about the health threats associated to the indoor use of e-cigs for both active and passive smokers.

## Figures and Tables

**Table 1:** Main characteristics of the coils used with the fourth-generation e-cig.

Coil	R (Ω)	Alloy	Wire Diameter ^1^ (mm)	Total Surface Area (mm^2^)	N. of Coils	N. of Aerosol Exit Holes	Power (W)	Heat Flux (kW m^−2^)
V8 Baby Q2	0.6	Kanthal	0.36	192	2	1	25	130
V8 Baby Q2	0.6	Kanthal	0.36	192	2	1	40	208
V8 Baby Q2	0.4	Kanthal	0.41	219	2	1	80	365
V8 Baby-T8	0.15	Kanthal	0.30	377	8	4	50	133
V8 Baby-T8	0.15	Kanthal	0.30	377	8	4	100	265
V8 Baby-T8	0.15	Kanthal	0.30	377	8	4	150	398

^1^ The wire diameter of each coil was measured with a high-precision caliber.

**Table 2:** Operative conditions and relevant codes of the experiments performed.

E-Cig	E-Liquid	Coil	Operative Conditions (W)	Code
First generation Young Category^®^	Liquid without nicotine	^1^	^1^	1st e-cig No Nic
	Liquid with a nicotine at 24 mg mL^−1^	^1^	^1^	1st e-cig Nic
Fourth generation G 150 Smok Kit^®^	Pacha Mama-Mango Pitaya Ananas^®^ without nicotine with 80/20 VG/PG	V8 Baby Q2 0.6 Ω	25	25 W No Nic 80/20 VG/PG
		V8 Baby Q2 0.6 Ω	40	40 W No Nic 80/20 VG/PG
		V8 Baby-T8 0.15 Ω	50	50 W No Nic 80/20 VG/PG
		V8 Baby Q2 0.4 Ω	80	80 W No Nic 80/20 VG/PG
		V8 Baby-T8 0.15 Ω	100	100 W No Nic 80/20 VG/PG
		V8 Baby-T8 0.15 Ω	150 ^2^	150 W No Nic 80/20 VG/PG
	SmookeKannel^®^ with nicotine at 9 mg mL^−1^ with 50/50 VG/PG	V8 Baby Q2 0.6 Ω	25	25 W Nic 50/50 VG/PG
		V8 Baby Q2 0.6 Ω	40	40 W Nic 50/50 VG/PG
		V8 Baby-T8 0.15 Ω	50	50 W Nic 50/50 VG/PG
		V8 Baby Q2 0.4 Ω	80	80 W Nic 50/50 VG/PG
		V8 Baby-T8 0.15 Ω	100	100 W Nic 50/50 VG/PG
		V8 Baby-T8 0.15 Ω	150 ^2^	150 W Nic 50/50 VG/PG

^1^ Coil already integrated in the cigarette, operative conditions are not settable by the vaper and not reported by the producers; ^2^ Beyond the interval indicated by the manufacturer.

## Data Availability

Data have been provided as tables and figures directly within the manuscript, and raw data are available via e-mail upon request to the corresponding author.

## Supplementary Materials

The following are available online at https://www.mdpi.com/article/10.3390/ijerph182111606/s1, Figure S1: Particle number concentrations averaged over the vaping sessions as functions of the heat fluxes. (**a**) Nicotine 50/50 VG/PG liquid, (**b**) Nicotine-free 80/20 VG/PG liquid, Figure S2: Particle volume concentrations averaged over the vaping sessions as functions of the heat fluxes. (**a**) Nicotine 50/50 VG/PG liquid, (**b**) Nicotine-free 80/20 VG/PG liquid, Figure S3: Particle size number (**a**) and size volume (**b**) distributions measured at 8, 10, 16, 24, 30 min time points of Figure 5.
