# Supplementary material for: Passive Vaping from Sub-Ohm Electronic Cigarette Devices
Source: Int J Environ Res Public Health. 2021 Nov 4;18(21):11606. doi: 10.3390/ijerph182111606 (PMC8583564; doi:10.3390/ijerph182111606)
Supplement: Supplementary file 1 [file ijerph-18-11606-s001.zip › ijerph-1324603-supplementary.pdf]

Passive Vaping from Sub-Ohm Electronic Cigarette Devices

Supplementary Materials

Maurizio Manigrasso [1,*], Carmela Protano [2,*], Matteo Vitali [2] and Pasquale Avino [3]

[1] Department of Technological Innovations, INAIL, Via IV Novembre 144, 00187 Rome, Italy
[2] Department of Public Health and Infectious Diseases, Sapienza University of Rome, P.le Aldo Moro, 5, 00185 Rome, Italy; matteo.vitali@uniroma1.it
[3] Department of Agricultural, Environmental and Food Sciences (DiAAA), University of Molise, via F. De Sanctis, 86100 Campobasso, Italy; avino@unimol.it
* Correspondence: m.manigrasso@inail.it (M.M.); carmela.protano@uniroma1.it (C.P.)

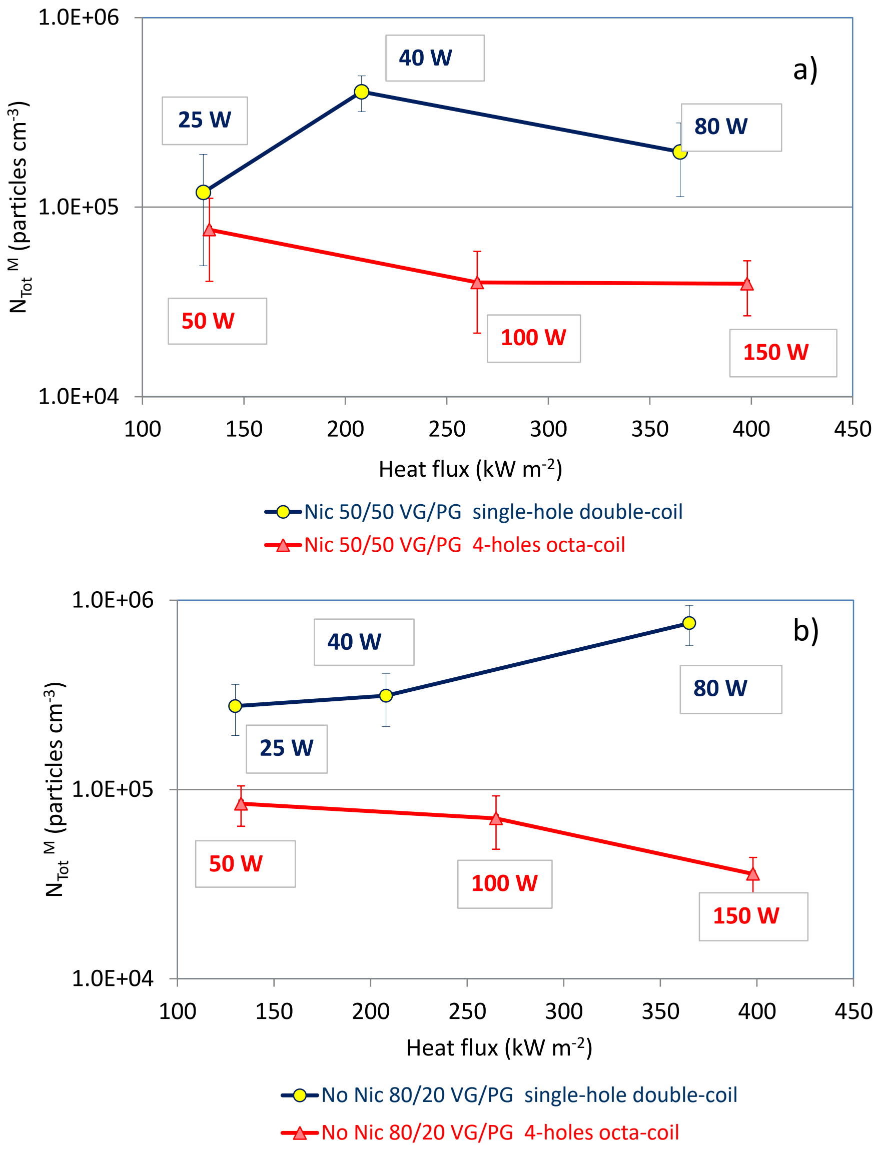

Figure S1. Particle number concentrations averaged over the vaping sessions as functions of the heat fluxes. (**a**) Nicotine 50/50 VG/PG liquid, (**b**) Nicotine-free 80/20 VG/PG liquid.

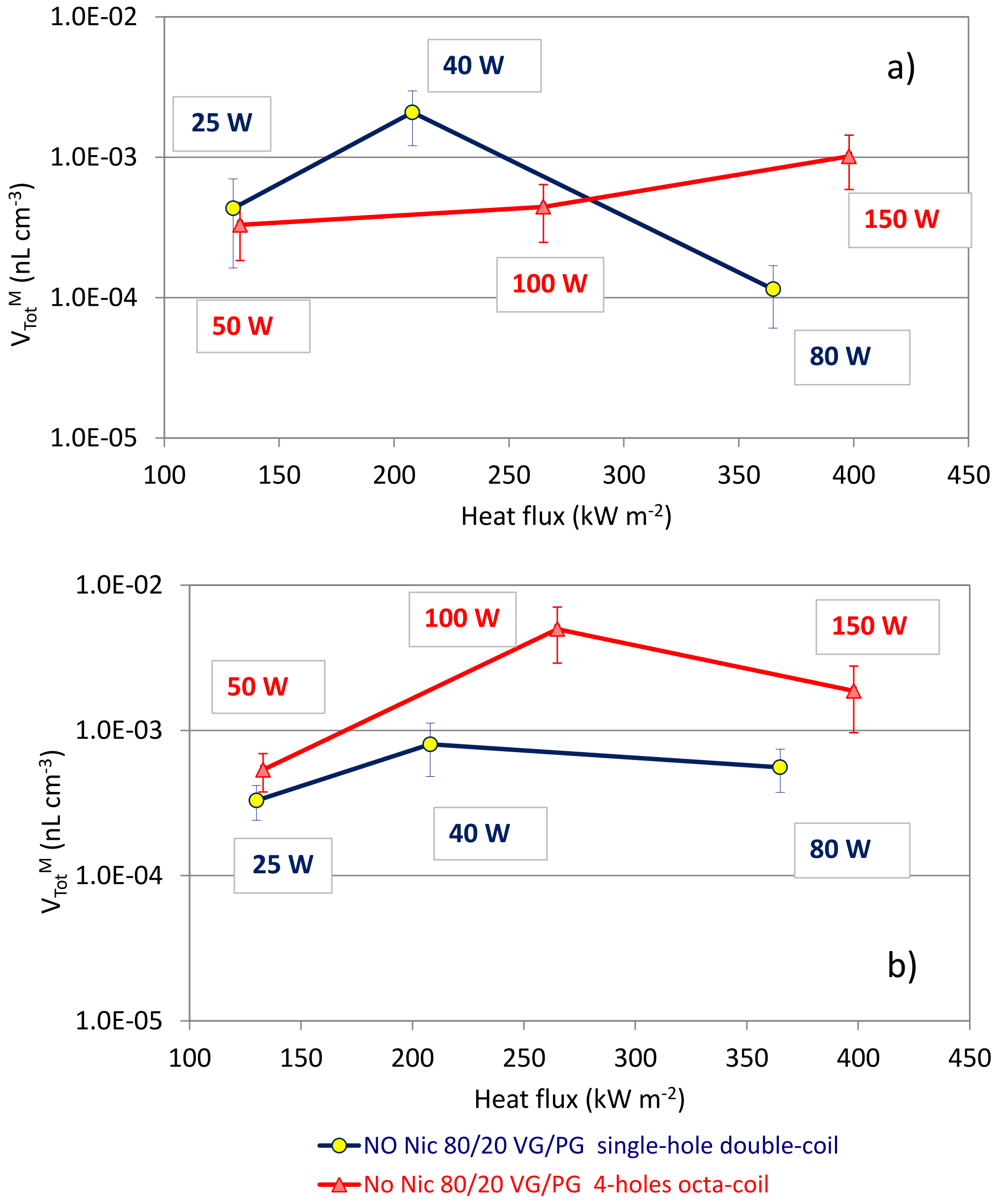

Figure S2. Particle volume concentrations averaged over the vaping sessions as functions of the heat fluxes. (**a**) Nicotine 50/50 VG/PG liquid, (**b**) Nicotine-free 80/20 VG/PG liquid.

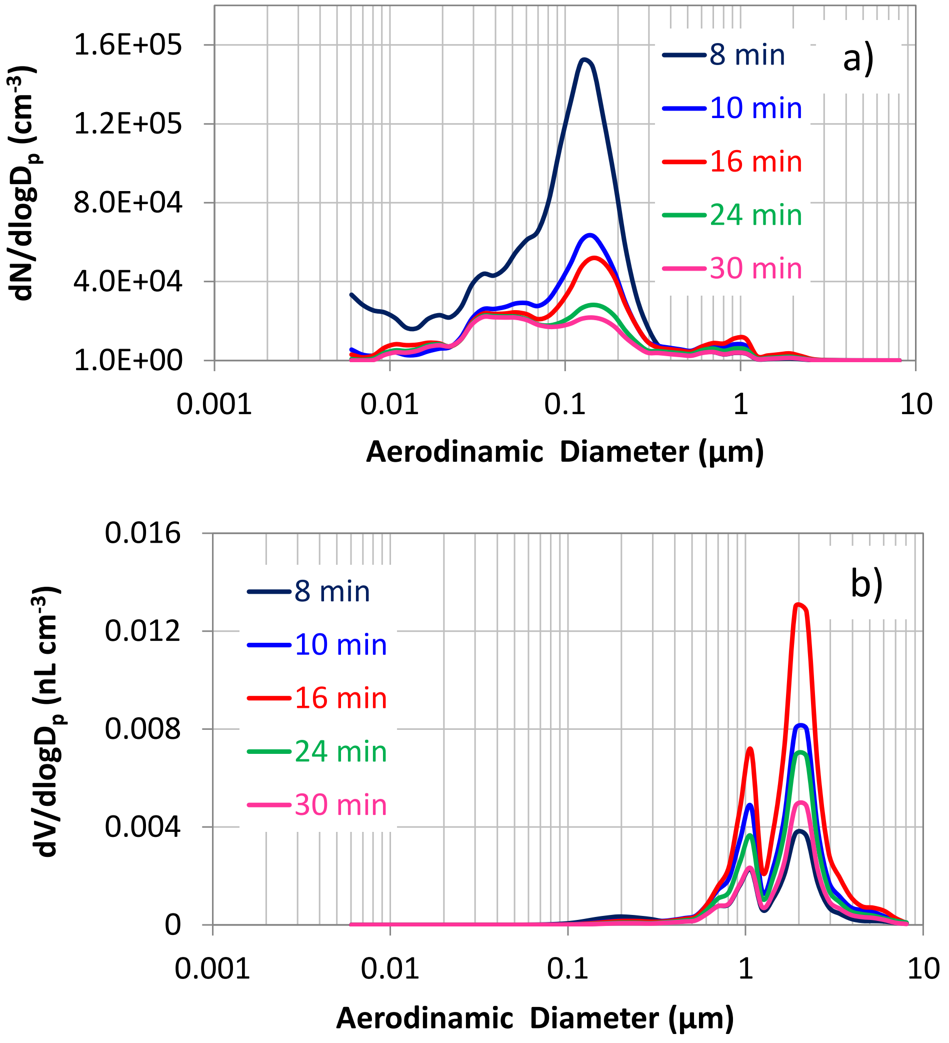

Figure S3. Particle size number (**a**) and size volume (**b**) distributions measured at 8, 10, 16, 24, 30 min time points of Figure 5.
